# Examining the impact of time management and resilience training on work-family conflict among Iranian female nurses: a randomized controlled trial

**DOI:** 10.1186/s12912-023-01634-w

**Published:** 2023-12-08

**Authors:** Sedigheh Peykar, Hakimeh Vahedparast, Tayebeh Gharibi, Razieh Bagherzadeh

**Affiliations:** 1 Bushehr University of Medical Sciences, Bushehr, Iran; 2 Nursing Faculty of Bushehr University of Medical Sciences, Bushehr, Iran; 3 Midwifery Faculty of Bushehr University of Medical Sciences, Bushehr, Iran

**Keywords:** Time management, Work-family conflict, Resilience training, Nurses

## Abstract

**Background:**

Female nurses confronting work-family conflict may endure adverse consequences for themselves and their families, leading to a decline in job performance and intentions to quit. Investigating the effects of interventions based on factors contributing to work-family conflict can aid in identifying optimal strategies for conflict reduction and mitigating its negative repercussions. This study aimed to examine the impact of time management and resilience training on work-family conflict among Iranian female Nurses in 2022.

**Methods:**

In this randomized controlled trial employing pre, post, and follow-up measures, 132 female nurses employed in the hospitals of Bushehr University of Medical Sciences (Bushehr, Iran) were selected and subsequently categorized into three groups—time management, resilience training, and control groups—using simple random sampling method. 10 online training sessions were conducted for each intervention group. A demographic form and a work-family conflict questionnaire were used for data collection. Repeated measures ANOVA, one-way ANOVA and multivariate general linear model were used to examine the hypotheses.

**Results:**

Regarding total work-family conflict, posttest mean scores were significantly lower than the pretest in both time management (*p* < 0.001) and resilience (*p* < 0.001) training groups, but follow-up mean scores were significantly higher than posttests in both time management (*p* < 0.001) and resilience (*p* < 0.001) training groups. In the control group, the mean scores at three time points were not statistically different (*P* = 0.058). The post-test mean score of work-family conflict was comparable in the two intervention groups (*P* > 0.05) and lower than the control group (*P* < 0.001) The follow-up mean score was comparable in the two intervention groups (*P* > 0.05) and lower than the control group (*P* < 0.001).

**Conclusion:**

Time management and resilience training effectively reduced the work-family conflict experienced by female nurses. Therefore, it is recommended that training programs such as time management and resilience training be incorporated into the ongoing education of nurses to alleviate their work-family conflict. Considering the diminishing impact of these interventions over time, training should be reiterated based on the evolving needs of the nurses.

**Trial registration:**

Number (IRCT20190122042453N2)*,*01/27/2022.

## Background

The rise of dual-career families has posed challenges for some women in managing the delicate balance between paid work and family responsibilities, resulting in work-family conflict (WFC) [1]. WFC is classified as a type of role conflict. The simultaneous existence of two or more role pressures generates psychological tension, making it challenging to engage in one role while fulfilling the demands of the other role [2, 3]. WFC encompassing two main types: interference of work with family and family with work.

Due to traditional gender roles, women are more susceptible to experiencing WFC than men [4]. Certain professions, such as nursing, are particularly prone to WFC due to unique professional conditions. A study in the United States revealed that 81% of new nurses reported experiencing WFC [5]. Similarly, a study in Iran found that 93% of nurses faced an average to high level of WFC [6]. Nurses encounter WFC due to factors like low organizational support, varying work shifts, demanding workloads, and specialized roles in healthcare areas such as intensive care units and emergency departments [7].

Nurses in Iran, a predominantly female profession, are especially vulnerable to WFC. The mandatory nursing hours are 175 hours per month, and due to a shortage of nurses, most work beyond these hours [8]. Female nurses often have at least two-night shifts per week, and the nature of the job, involving physical and psychological pressure, compounds their challenges [8, 9]. Family-related responsibilities, such as caring for children, cooking, cleaning, and tending to spouses or elderly family members, add additional pressure, contributing to WFC [4].

WFC has been associated with adverse effects on physical and mental well-being, manifesting as poor health, symptoms of depression, high blood pressure, obesity, increased blood cholesterol, sleep problems, anxiety, altered organizational attitudes, reduced job satisfaction, diminished organizational commitment, disruptions in job performance, intentions to quit, absenteeism, negative attitudes towards family members, decreased life satisfaction, and reduced marital satisfaction [10–12].

According to Greenhouse and Beutel’s model, WFC can originate from three sources: time, behavior, and pressure. Time-based conflict arises when work demands significant time allocation, behavioral conflict emerges when behavioral expectations differ between work and family, and pressure-based conflict occurs with increased pressure in a specific area [13]. Work-related pressures and time constraints, resulting in the loss of resources like time and energy, are among the primary causes of WFC. The Conservation of Resources Theory (COR) suggests that individuals, including nurses, seek resources to cope with stress [14]. Addressing this resource loss through interventions can help alleviate the stress induced by WFC.

Time-based WFC suggests that learning optimal time management methods can potentially reduce WFC [15]. Given that nursing requires multitasking and dealing with time constraints and work-related pressures, nurses should acquire time management skills and strategies to better balance their work and family roles [16, 17].

Despite the importance of understanding interventions that reduce WFC and its consequences, few studies have explored the effects of interventions on predictors or consequences of WFC. A study by Rasooli et al. (2009) demonstrated that time management training in nurses reduced WFC [18]. An interventional study on hospital emergency technicians showed that time management training reduced WFC. Khosravan et al. (2018) found that a family-oriented support package, including time management and stress management, reduced WFC among nurses working in Zabol, Iran [19]. One study found that a group treatment intervention tailored to the needs of healthcare professionals had the potential to improve self-efficacy and work-life balance [20].

Resilience, considered a resource, plays a role in coping with the pressures associated with nursing jobs [21]. Studies have demonstrated an inverse relationship between resilience as part of psychological capital and WFC [22]. Resilience training has been shown to reduce anxiety and stress in nurses, highlighting its potential to address the relationship between WFC, resilience, and the learnability of resilience [23]. Several studies have explored the effects of stress-reducing interventions, such as mindfulness, on WFC. However, the results have been contradictory [24–26]. Additionally, some studies suggest that developing resilience can reduce stress and anxiety while increasing self-efficacy [27–30]. Nevertheless, an investigation of existing literature did not reveal any studies that specifically explored the impact of resilience on WFC. Furthermore, no study has been identified that compares the effects of teaching time management and resilience on WFC. Such comparisons can contribute to identifying the most effective interventions and subsequently reducing WFC.

Therefore, this study aims to examine the impact of time management and resilience training on WFC among female nurses working in all hospitals of Bushehr University of Medical Sciences (southern Iran) during 2022.

### Theoretical framework

The present study is grounded in the WFC theory proposed by Greenhouse and Beutel (1985). According to this theory, WFC phenomenon has three origins, encompassing conflict based on time, behavior, and pressure [13, 31]. The model posits that when the work area demands substantial time allocation, WFC arise based on time [31]. Factors such as long working hours, job inflexibility, and shift work contribute to time-based interference between work and family [32]. In the family domain, various factors, including having young children, a working spouse, and numerous family members, lead to extensive time devoted to the family, thereby causing interference of the family domain with the time-based work domain [4].

Pressure-based conflict arises when pressure intensifies in a particular domain. For instance, ambiguity in the work role, conflict within the work area, and the broad scope of activities increase pressure in the work domain, resulting in interference of the work area with the family. Conversely, factors such as conflict within the family and inadequate support from the spouse elevate pressure in the family domain, leading to interference of the family with work dimensions in a pressure-based manner [31].

Behavioral conflict emerges when behavioral expectations in the work and family domains differ for an individual. In the work domain, individuals are expected to be reserved and objective, while in the family domain, warmth and emotion are expected. This incongruity in behavioral expectations gives rise to behavior-based conflict [31, 33].

Another foundational theory underpinning this research is the Conservation of Resources Theory (COR). This theory posits that individuals strive to conserve and acquire resources to minimize and cope with stress. Deprivation or removal of these resources induces stress. Resources, in this context, encompass objects, conditions, personal characteristics, and energy [14].

### Conceptual framework and hypothesis

**Fig. 1:**
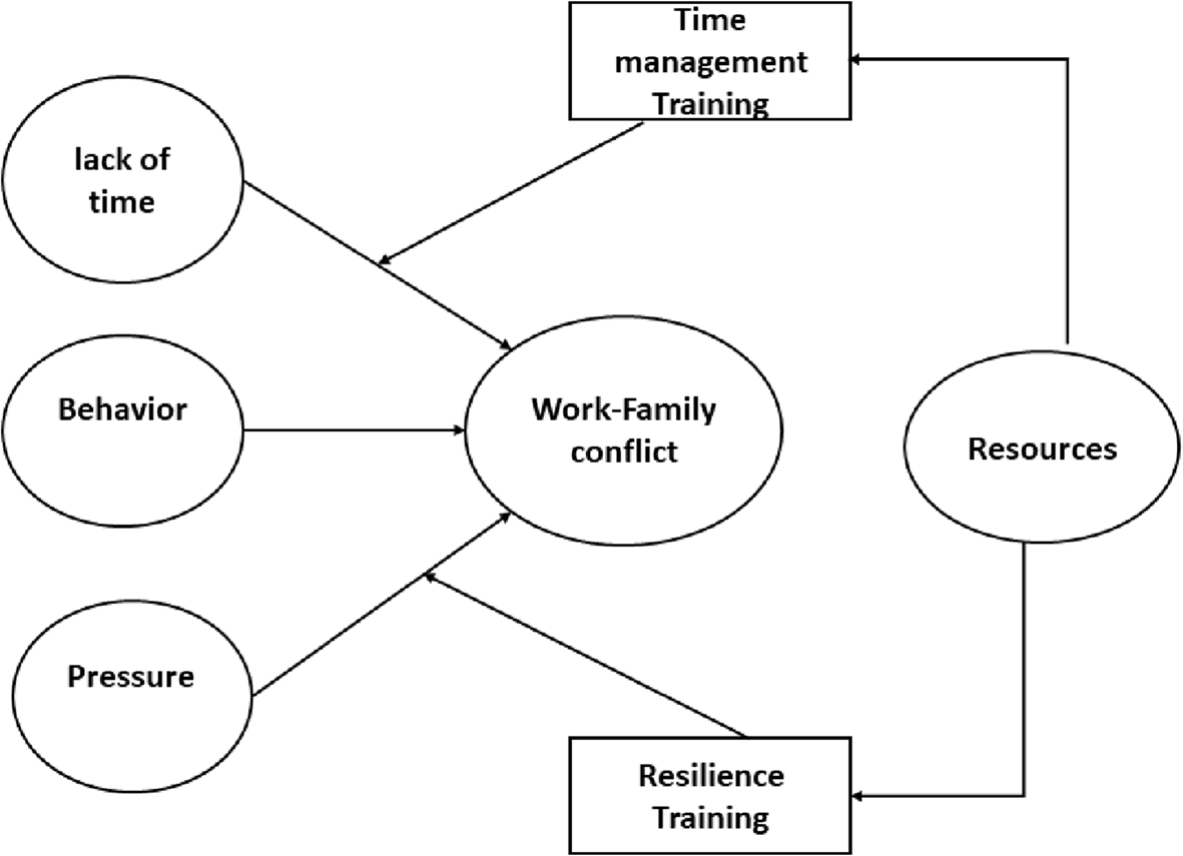
Conceptual frameworke

According to the WFC theory proposed by Greenhouse and Beutel, WFC originates from three sources: time, behavior, and pressure [13, 31]. Balancing the demands of work and family can lead individuals to perceive a loss of resources, such as time and energy, resulting in stress [34]. Drawing from the Conservation of Resources Theory (COR), individuals, to cope with stress, need to acquire resources such as time, self-confidence, self-efficacy, motivation, and energy [14]. In the conceptual framework of this study, effective time management and building resilience emerge as pivotal strategies to alleviate stress arising from conflicts between work and family responsibilities. The conceptual framework is illustrated in Fig. 1. Based on this conceptual framework, three hypotheses are proposed for the current research:

*Hypothesis 1*: Time management training leads to a reduction in WFC.

*Hypothesis 2*: Resilience training leads to a reduction in WFC.

*Hypothesis 3*: Time management and resilience training have a similar effect in reducing WFC.

## Materials and methods

### Study design and population

This study employed an experimental design, specifically a randomized controlled trial with three parallel arms (two intervention groups and one control group). The study encompassed pre-test, post-test, and follow-up assessments and targeted female nurses employed in all hospitals affiliated with Bushehr University of Medical Sciences, Bushehr, Iran, in 2022. Inclusion criteria comprised female nurses working in clinical departments of Bushehr University of Medical Sciences hospitals, married and cohabiting with a spouse, a minimum of 6 months of nursing work experience, willingness to participate in the study, and obtaining a score above the median on the WFC questionnaire. Exclusion criteria encompassed previous participation in time management and resilience workshops, self-reported use of psychiatric medication, self-reported mental illness, transfer to a non-nursing department or a city outside the province, long-term sick leave, and experiencing a stressful event (e.g., death of relatives, severe illness in close relatives) in the last 6 months.

### Sample size and sampling methods

Following the study by Rasooli et al. (2009), the mean and standard deviation of WFC in the group receiving time management and control after the intervention were 40.19 ± 13.65 and 54.65 ± 15.91, respectively [18]. Considering a type 1 error of 0.05, a power of 95%, and a 10% dropout probability, the sample size was determined to be 40 individuals per group. A total of 275 nurses were screened, with 175 meeting the inclusion criteria. From these, 132 participants agreed to take part in the study and were randomly assigned to one of the three study groups consisting of 44 participants per group (i.e., resilience training, time management training, and control) using simple randomization (Allocation ratio: 1/1/1). Random allocation software was employed, and the list of phone numbers of eligible nurses was given to an unfamiliar person for the randomization process. The randomization list was concealed until shortly before the intervention.

**Fig. 2:**
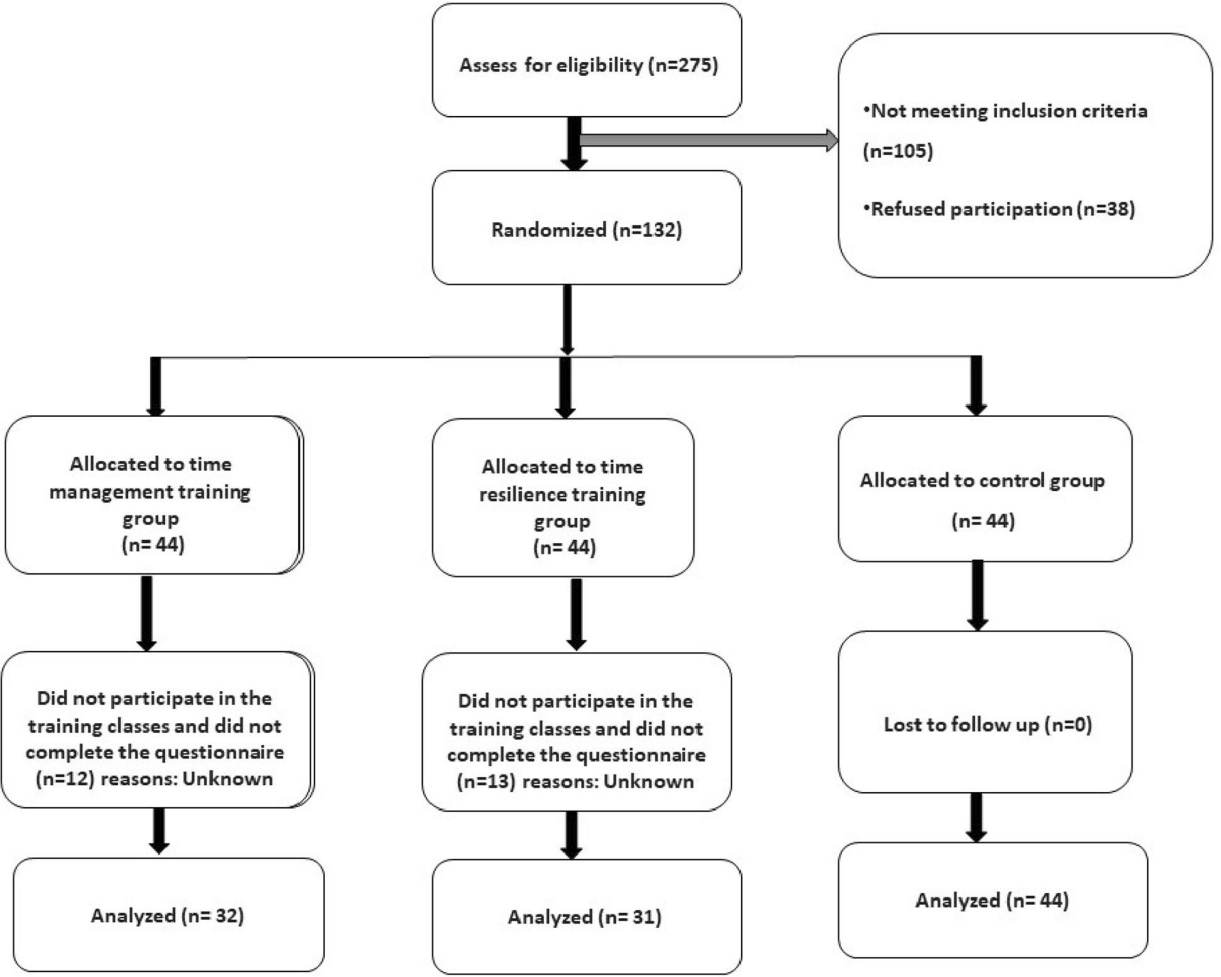
Consort diagram

After the intervention, 13 individuals from the resilience group and 12 from the time management group did not participate in the training classes and did not complete the questionnaire. However, all the participants in the control group completed the questionnaire at three different times, resulting in a final analysis of 107 individuals. The Consort flowchart is presented in Fig. 2.

### Research implementation method

The study protocol received approval from the Research Ethics Committee of the Vice-Chancellor of Bushehr University of Medical Sciences, Bushehr, Iran. After obtaining ethical approval and registration in the clinical trial system (IRCT20190122042453N2), written permission was obtained from officials of the hospitals affiliated with Bushehr University of Medical Sciences, Iran. Sampling recruitment commenced on 10-2-2022, and the intervention was conducted from 02 to 04-2022 to 30-06-2022. The principal investigator went to the hospitals, communicated with nurses at the end of each work shift, and explained the research objectives to eligible nurses. Those willing to participate completed a demographic form and WFC questionnaires, providing contact information for the subsequent part of the study if they met the inclusion criteria (i.e., a score above the median in the WFC questionnaire). Simple random allocation placed the participants into three groups: time management training, resilience training, and control.

During an online meeting via the Big Blue Button platform, the participants in the two intervention groups were briefed about the study objectives and how it was going to be conducted. The necessary arrangements were made via phone, and meeting links were shared through WhatsApp. Two WhatsApp groups were created for the intervention groups, and written informed consent was obtained. In the control group, consent forms were sent privately to each participant on WhatsApp. To balance training time and goals, each intervention group was divided into two for online sessions. 10 sessions of time management and resilience training, one per week (1 hour and 45 minutes each), were conducted online through the Big Blue Button platform, accommodating the shift work of most nurses. The recorded files of the sessions were shared in the WhatsApp groups. Training was delivered by an individual with expertise in nursing and an academic degree in clinical psychology (the third author) and an M.Sc. student in psychiatric nursing (the first author). Follow-up exercises that were part of the training were managed through the WhatsApp group. In addition, the participants could report privately if they were unwilling to share their personal matters.

The time management training package comprised 10 sessions based on Brian Tracy’s principles, previously applied by Zaerian et al. (2007) on education staff in Iran [35]. Adaptations were made for nursing conditions, with validation by one expert with a doctorate degree in clinical psychology and another expert in the field of management. The resilience skill training package, also with 10 sessions, drew on literature review and expert opinions [36, 37], tailored to nursing and the challenges of balancing work and family duties. In addition, the content validity was confirmed by two psychologists. A summary of the training model, developed and implemented based on the ADDIE (Analysis, Design, Development, Implementation, and Evaluation) model, is shown in Table 1. Tables 2 and 3 outlines the titles of resilience and time management training sessions.

**Table 1:** ADDIE (Analysis, design, development, implementation, and evaluation) model

Analysis	Needs assessment through searching on reputable websites.
	Determining a valid questionnaire for measuring work-family conflict.
	Literature review to find the best solutions.
	Establishing general objectives based on time management and resilience.
Design	Setting specific goals based on time management and resilience.
	Determining the educational content for each session based on time management and resilience.
	Identifying the educational strategy (lectures and group discussions).
	Determining the media/platforms to be used (PowerPoint, BigBlueButton, WhatsApp, instructional videos).
Development	Determining the time framework for each session (each session: presentation and group discussion for 60 minutes, reviewing assignments and providing feedback for 45 minutes).
	Organizing content based on the media to be used.
Implementation	Presentation of educational content on time management and resilience.
	Assignment presentation related to each session.
Evaluation	Evaluation is performed in two forms: formative and final. Formative Evaluation: Examining the tasks of each session and providing feedback. Evaluation includes sharing experiences in two separate WhatsApp groups and discussing them. Final Evaluation: Completing the work-family conflict questionnaire. Sharing experiences in two separate WhatsApp groups and discussing them.

**Table 2:** Time management training sessions

	Topics	Assignments
First session	Introduction, creating motivation, reviewing the structure of the meetings and the main rules, setting goals, and planning training.	Listing 10 goals for the coming year. Writing down the activities of the next 24 hours.
Second session	Teaching the 80/20 rule, the concept of considering the consequences of actions, resistance to education and change	Preparing a list of all activities based on the 80/20 rule. Identifying potential resistances to task completion.
Third session	Teaching the A, B, P, T, S method of focusing on goals	Selecting an activity based on criteria A, B, P, T, S, and identifying strengths and weaknesses to achieve goals.
Fourth Session	Understanding the principle of recognizing necessity and emphasizing the importance of making preparations before commencing work.	Visualizing the workplace to identify the necessity of a suitable environment for the activity.
Fifth Session	Understanding the importance of inclusiveness within one’s profession on a permanent basis and the importance of unique talents	Identifying unique and positive aspects of your existence.
Sixth session	Understanding the limiting factors of progress and emphasizing the concept of breaking down large tasks into smaller parts.	Drafting important life goals and providing a sample breakdown of tasks into smaller sections for execution.
Seventh session	Understanding the significance of setting time limits for tasks and recognizing the importance of concentrating physical and mental efforts on a specific subject.	Developing an activity plan based on time, energy, sleep and eating habits, and controlling thoughts.
Eighth session	Understanding positive internal conversations and their impact on performance, as well as becoming acquainted with the concept of constructive laziness.	Controlling thoughts, constructive laziness.
Ninth session	Understanding the importance of avoiding difficult tasks and becoming familiar with methods to increase available time through the implementation of time management strategies.	Creating a list of activities and visualizing the most challenging task along with methods of accomplishing it.
Tenth session	Becoming acquainted with the concept of enhancing action speed through the implementation of time management skills, and understanding the significance of focusing on one task at a time.	Selecting an activity that needs to be completed quickly.

**Table 3:** Resilience training sessions

	Topics	*Assignments*
First session	Familiarity: communicating with the audience and familiarizing them with resilience and the rules of participation in the workshop	List of actions to overcome daily stresses.
Second session	Self-awareness/ awareness of one’s capabilities	Describing general features and characteristics, beliefs, values, thoughts, feelings, and personal goals.
Third session	Volubility/ strengthening of self-esteem	Presenting an experience of the impact of self-esteem on life.
Fourth Session	Effective communication and bonding / improving people’s ability to communicate/establishing social relations and making friends	Sharing concrete experiences of your friendships along with the advantages and disadvantages of those relationships.
Fifth Session	Foresight/determining the goal and how to achieve it	Setting short-term and long-term goals on the worksheet.
Sixth session	Self-efficacy/ decision making	Listing decisions made in the past week along with their outcomes.
Seventh session	Self-efficacy/ problem solving	Describing the experience of the problem-solving stages.
Eighth session	Self-efficacy/ responsibility	Delegating a task to a spouse, child, or accepting a new responsibility.
Ninth session	Controlling emotions/ managing anger, anxiety and stress	Performing a weekly reflection.
Tenth session	Meaningfulness/ Cultivating a sense of spirituality and faith	Expressing the difference between optimism, realism, and pessimism.

Posttest and one-month follow-up assessments (1 month later) involved both intervention and control groups completing the WFC questionnaire online. Questionnaire links were sent to the WhatsApp group for the intervention groups and privately to the control group.

Due to the study’s nature, blinding was not feasible. Steps were taken to minimize performance bias, including separate WhatsApp groups for each intervention group, coordination with head nurses for at-home participation, and providing questionnaire links through WhatsApp for post-test and follow-up data collection. An individual not involved in the research entered completed questionnaires into statistical software, i.e., SPSS, reducing bias. Be that as it may, the authors acknowledge the potential for bias, as virtual union groups exist to which all nurses of the province have subscribed.

### Measures

The outcome measured was WFC, assessed using data collected through a demographic and occupational form and a WFC questionnaire. The demographic and occupational form gathered information on age, marriage age, working hours per week, number of night shifts per month, work experience, number of children, age of the youngest child, economic status of the family, age, education level, and job of the spouse.

The WFC questionnaire for Iranian married women was developed by Bagherzadeh et al. (2016), comprising 37 items across four domains. Domain 1, “work role interference with personal and family life,” consists of 17 items (items 9 to 24 and item 29). An example item is “I devote a small amount of time for my family”. Domain 2, “opposition and dissatisfaction of the family,” includes 7 items (items 25 to 28 and 1, 2, and 8). An example item is “ Family members are always complaining about my absence”. Domain 3, “the interference of the family role with the work role,” comprises 8 items (items 30 to 37). An example item is “ I get to work with delay or leave the work earlier”. Domain 4, “inadequate facilities and support,” involves 5 items (items 3 to 7). An example item is “ I can count on the help of people around me to coordinate my job and family”. Response options range from strongly agree to strongly disagree, as well as reverse-scored questions (items 3, 4, 5, and 6). The scores for each domain and total WFCscores were converted into percentage scores through linear transformation, resulting in scores ranging from 0 to 100. Bagherzadeh et al. (2018) confirmed the face validity, content validity, structure, and reliability of the instrument among women in Bushehr province. The face validity of the items was established with an item impact score ranging from 2.8 to 5. The average content validity index was 0.98, indicating the tool’s appropriate content validity. The Cronbach’s alpha for the entire questionnaire was 0.92, and the intra-class correlation coefficient between test and retest was 0.98, demonstrating the tool’s satisfactory reliability [38]. In the present study, Cronbach’s alpha for the entire questionnaire was 0.90, 0.93, and 0.91 at pretest, posttest, and follow-up, respectively. The range of Cronbach’s alpha for the work-family domain at three time points was between 0.87 and 0.94.

### Data analysis

Data analysis utilized SPSS software (version 19). Descriptive statistics (frequency, mean, and standard deviation) were reported after refining the data, ensuring no outliers or missing data in the main variables. The distribution of data was assessed using the Shapiro-Wilk Test. One-way analysis of variance or the Kruskal-Wallis test, along with chi-square or Fisher exact test, compared demographic and occupational characteristics between the three groups.

Repeated measures ANOVA assessed the mean WFC and its domains between pre-test, post-test, and follow-up for the three groups, with LSD post hoc tests for two-by-two comparisons.

Between-group comparisons analyzed the mean score between the three groups at the three time points using multivariate GLM (General Linear Model) for WFC domains and one-way analysis of variance for total WFC. LSD post hoc tests were employed for two-by-two comparisons. Multivariate GLM included mean WFC domains and groups as dependent and independent variables, respectively. The significance level was set at < 0.05 in all analyses.

## Results

Out of the 132 participants, 13 from the resilience group and 12 from the time management group did not participate in training classes and did not complete the post-test and follow-up questionnaires. All the participants in the control group completed the questionnaire at all three assessment points, resulting in a final analysis of 107 individuals (Fig. 2). Dropout analysis revealed no significant differences between those who completed post and follow-up measures and those who only completed the pre-measure in terms of demographic, occupational, and pre-test variables.

The average age of the participants in the time management, resilience, and control groups was 32.06 ± 5.40, 33.29 ± 4.78, and 33.48 ± 6.09, respectively. Tables 4 present a comparison of quantitative and qualitative demographic variables between the three groups. Statistical tests indicated no significant differences among the three groups in terms of demographic and occupational variables.

**Table 4:** Comparison demographic and occupational variables between three groups of time management training, resilience training and control

Variable		Group			Statistic (*P*value)
		Time management	Resilience	Control	
		Mean ± SD or N(%^e^)	Mean ± SD or N(%^e^)	Mean ± SD or N(%^e^)	
Age/year		32.06 ± 5.40	33.29 ± 4.78	33.48 ± 6.09	0.666 ^a^ (0.516)
Marriage age		20.27 ± 8.82	18.93 ± 7.92	20.21 ± 8.37	0.251 ^a^ (0.779)
Clinical experience/month		98.58 ± 70.81	106.07 ± 64.41	109.66 ± 75.34	0.223 ^a^ (0.800)
Working hours per week		59.86 ± 21.57	52.92 ± 14.68	61.87 ± 39.11	0.767 ^a^ (0.467)
Number of night shifts per month		5.90 ± 5.04	5.79 ± 4.84	4.76 ± 3.25	0.775 ^a^ (0.463)
Number of children		1.13 ± .85	1.37 ± .85	1.29 ± 0.92	1.131^b^ (0.568)
Age of the youngest child/month		37.03 ± 42.33	40.83 ± 29.96	48.90 ± 52.20	0.6969 ^a^ (0.501)
Spouse’s age/years		33.37 ± 7.88	35.63 ± 4.80	36.02 ± 5.86	1.717 ^a^ (0.185)
Husband’s university education	No	8(25.8)	4(13.3)	6(14.0)	2.234^c^ (0.332)
	Yes	23(74.2)	26(86.7)	37(86.0)	
Husband’s job	Employee	19(63.3)	19(65.5)	33(76.7)	3.982^d^ (0.472)
	Manual worker	4(13.3)	3(10.3)	1(2.3)	
	Retired	7(23.3)	7(24.1)	9(20.9)	
Economic situation	Good	20(66.7)	23(52.3)	16(50.0)	2.997^d^ (0.563)
	Moderate	7(23.3)	17(38.6)	11(34.4)	
	Poor	3(10.0)	4(9.1)	5(15.6)	
Department of service	Critical care unit	2(7.1)	6(20.7)	10(23.8)	4.416 ^d^ (0.372)
	Pediatric or neonatal ward	5(17.19)	4(13.8)	1(2.4)	
	Operating room	4(14.3)	4(13.8)	7(16.7)	
	Emergency ward	9(31.1)	6(20.7)	9(21.4)	
	General ward	8(28.6)	9(31.0)	15(35.7)	

^a^The one-way analysis of variance was performed and the reported statistic is F values; ^b^The Kruskal-Wallis test was performed and the reported statistic is X^2^ values; ^c^ The Chi square test was performed and the reported statistic is X^2^ values; ^d^ The Fisher exact test was performed; ^e^ Percentages are valid percent

*N* Number, *SD *Standard deviation

The results of within-group comparisons revealed a significant difference between the three time points (pretest, posttest, and follow-up) concerning the mean score of WFC and its domains in both time management and resilience training groups. In the control group, within-group differences were significant for two domains (work role interference with personal and family life and family dissatisfaction) of WFC. The mean scores of the inadequate facilities and support domain were not statistically different between the three time points in all the three groups (Table 5).

**Table 5:** Comparison the mean scores of work-family conflict and its domains between three groups (time management training, resilience training and control) and between three times (Pretest, posttest and follow up)

Variable	Group	Time			Comparison of three times**
		Pre-test	Post-test	Follow-up	F (*P*value)
		Mean ± SD	Mean ± SD	Mean ± SD	
Work role interference with personal and family life	Time management training	63.23 ± 19.16	40.74 ± 17.24	50.23 ± 17.49	113.322 (< 0.001)
	Resilience training	65.05 ± 17.08	38.14 ± 13.72	49.41 ± 15.01	133.709 (< 0.001)
	Control	65.29 ± 16.02	55.67 ± 13.81	61.14 ± 14.66	12.759 (< 0.001)
Comparison of three groups*	F (*P* value)	0.146 (0.824)	15.579(< 0.001)	6.791 (0.002)	–
Family dissatisfaction	Time management training	50.38 ± 16.35	35.29 ± 16.79	44.04 ± 17.88	47.698 (< 0.001)
	Resilience training	50.91 ± 16.44	37.34 ± 15.14	45.90 ± 13.95	45.420 (< 0.001)
	Control	51.46 ± 17.50	45.20 ± 15.03	48.29 ± 15.07	10.121 (< 0.001)
Comparison of three groups*	F (*P* value)	0.039 (0.962)	4.351 (0.015)	0.700(0.499)	–
Family role interference with work role	Time management training	42.95 ± 27.51	27.34 ± 26.72	33.93 ± 25.66	30.544 (< 0.001)
	Resilience training	43.89 ± 19.32	28.30 ± 22.85	35.84 ± 22.66	29.112 (< 0.001)
	Control	40.06 ± 22.88	39.79 ± 21.26	41.74 ± 20.58	1.575(0.075)
Comparison of three groups*	F (*P* value)	0.278 (0.758)	3.390 (0.037)	1.232 (0.296)	–
Inadequate facilities and support	Time management training	61.72 ± 20.42	60.95 ± 17.13	58.47 ± 18.63	1.336 (0.270)
	Resilience training	63.22 ± 20.80	58.71 ± 17.12	57.26 ± 17.76	1.500 (0.064)
	Control	56.13 ± 16.52	57.90 ± 13.99	57.30 ± 15.97	0.877 (0.415)
Comparison of three groups*	F (*P* value)	1.482 (0.232)	0.350 (0.705)	0.052 (0.949)	–
Total work-family conflict	Time management training	54.57 ± 12.38	41.08 ± 10.83	46.67 ± 10.52	89.265 (< 0.001)
	Resilience training	55.77 ± 8.31	40.62 ± 7.08	47.10 ± 7.71	104.270 (< 0.001)
	Control	53.23 ± 10.66	49.64 ± 9.16	52.12 ± 9.39	1.542 (0.058)
Comparison of three groups*	F (*P* value)	0.526 (0.592)	11.898 (< 0.001)	4.124 (0.019)	–

* The analysis of Covariance was performed

**Repeated measure analysis of variance was performed

*SD* Standard deviation

**Fig. 3:**
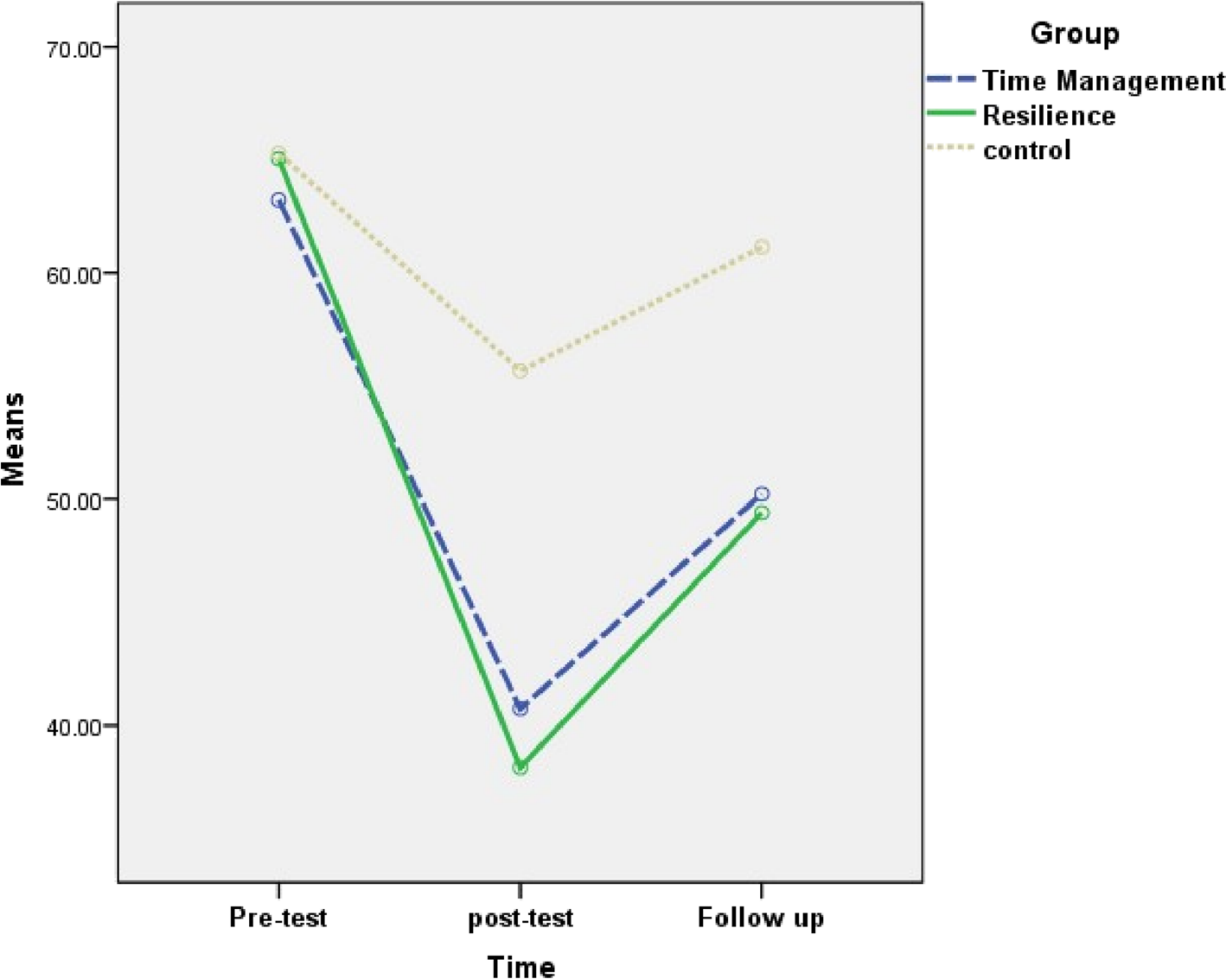
Changes of work role interference with personal and family life in three groups

**Fig. 4:**
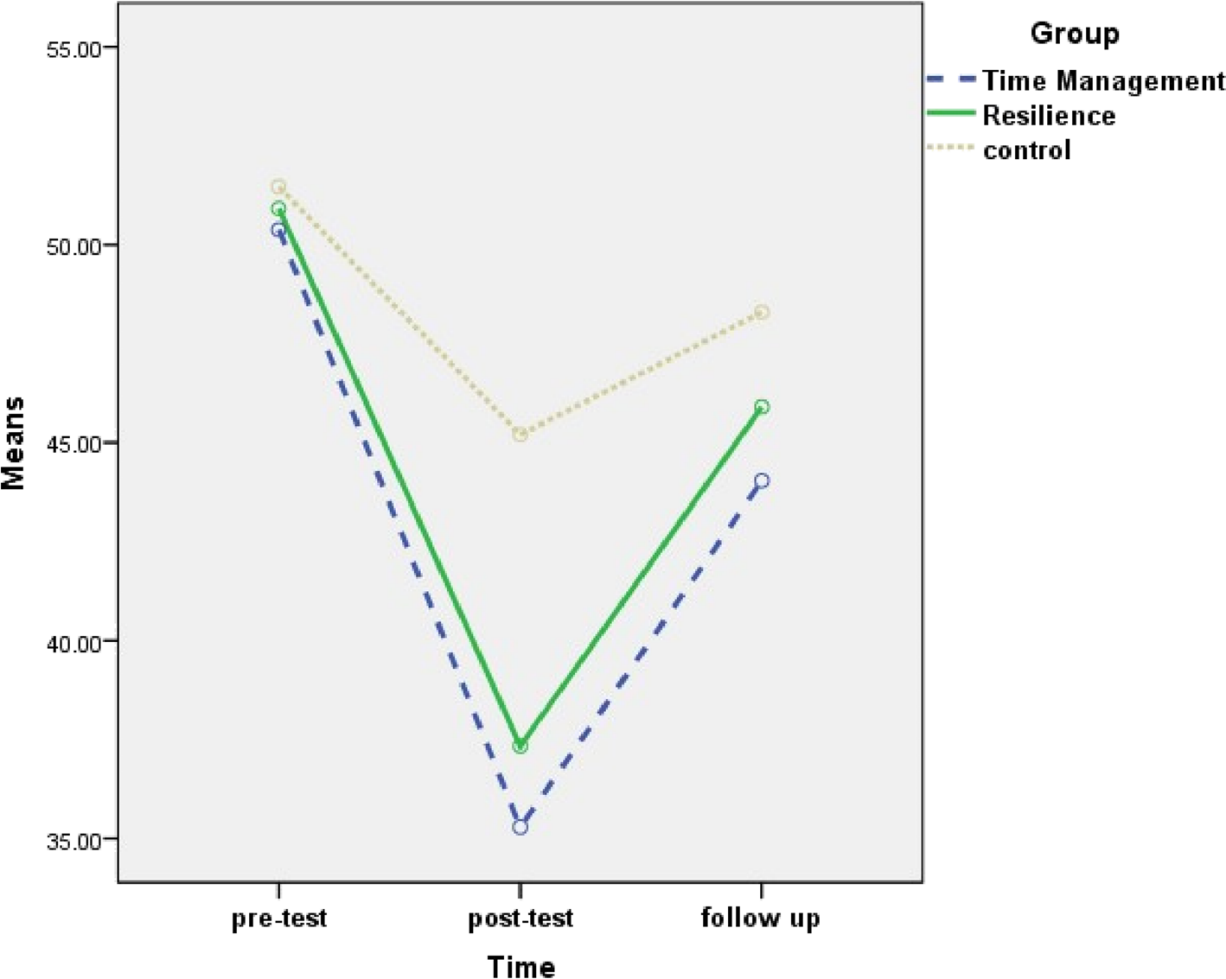
changs of family dissatisfaction in three groups

**Fig. 5:**
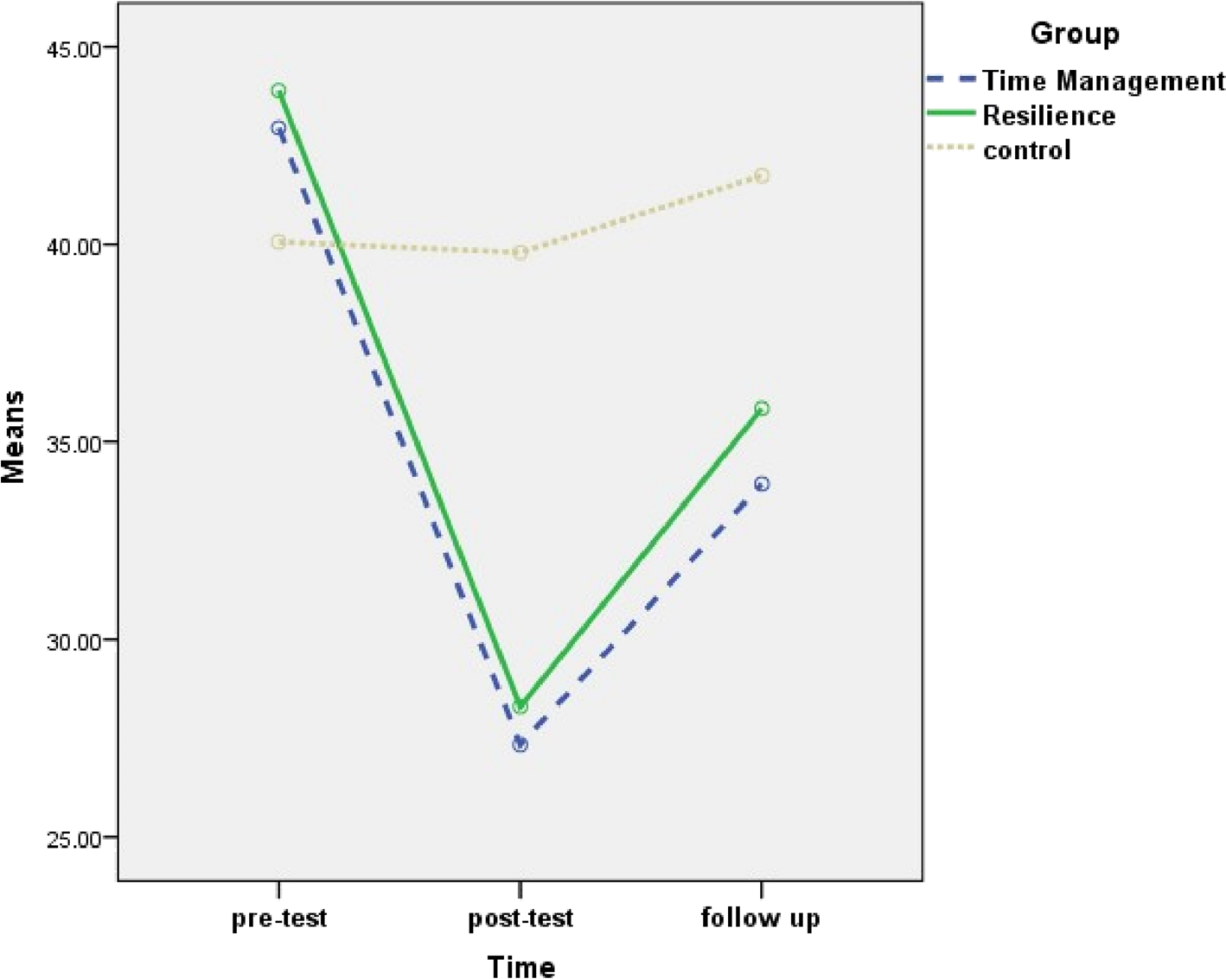
Changes of interference of family role with work rolein three groups

**Fig. 6:**
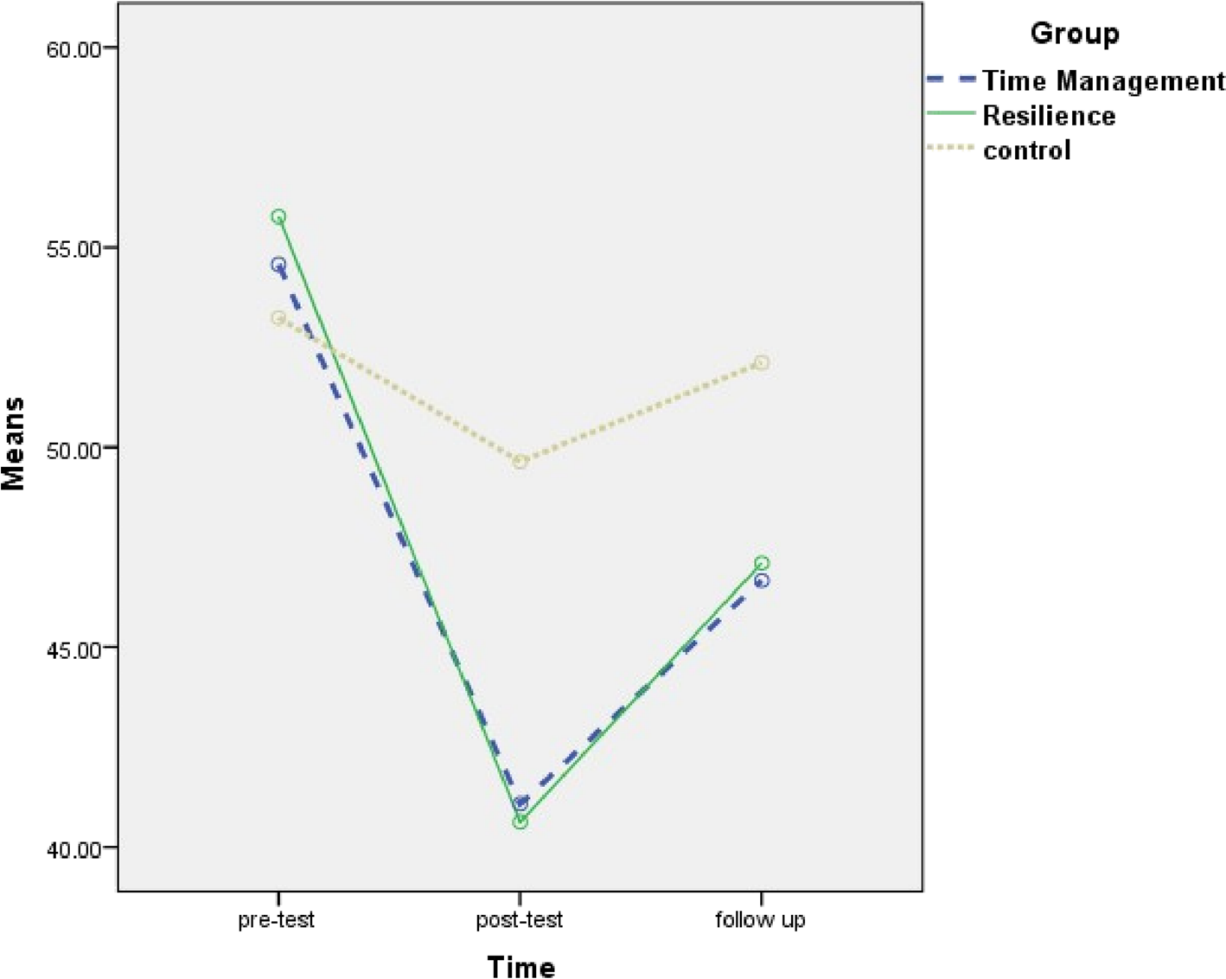
Changes of total work-family conflict in three groups

Two-by-two comparisons of the mean and standard deviation of the WFC score and its domains showed that, in the work role interference with personal and family life domain and the family dissatisfaction domain, the mean score of posttests and follow-up was significantly lower than the pretest, and the mean score of follow-up was significantly higher than posttest in all the three groups. In the family role interference with work role domain and total WFC, the mean score of posttests and follow-up was significantly lower than the pretest, and the mean score of follow-up was significantly higher than posttest in the two intervention groups (Table 6 and Figs. 3, 4, 5 and 6).

**Table 6:** Mean difference and confidence interval for two-by-two comparison of work-family conflict and its domains between three times (pretest, posttest and follow up) for time management training, resilience training and control groups

Variable	Group	Time		
		Pre-test and post-test	Pre-test and follow-up	Follow-up and post-test
		Mean difference (95% CI)	Mean difference (95% CI)	Mean difference (95% CI)
Work role interference with personal and family life	Time management training	−22.49 (− 26.158; −18.81)***	−13.00 (− 15.57; −10.43)***	9.49 (6.67; 12.31)***
	Resilience training	−26.91 (−30.13; − 23.70)***	− 15.65 (− 19.52; −11.78)***	11.26 (8.29; 14.24)***
	Control	− 9.63 (−14.37; − 4.88)***	−4.16 (− 7.69; − 0.62)*	5.47 (2.38; 8.56)**
Family dissatisfaction	Time management training	− 15.09 (− 18.41; − 11.76)***	−6.34 (− 9.81; − 2.88)**	8.75 (6.11; 11.38)***
	Resilience training	− 13.57 (−16.77; − 10.37)***	− 5.01 (− 8.14; − 1.89)**_	8.56 (6.12; 11.00)***
	Control	− 6.26 (− 9.29; − 3.23)***	−3.17 (− 6.25; − 0.10)*	3.09 (0.85; 5.33)**
Family role interference with work role	Time management training	−15.28 (− 20.40; − 10.15)***	−9.65 (− 13.81; − 5.48)***	5.63 (3.28; 7.98)***
	Resilience training	− 16.17 (− 21.02; − 11.33)***	− 8.83 (− 12.11; − 5.56)***	7.34 (2.60; 12.08)**
Total work-family conflict	Time management training	−13.49 (− 16.11; − 10.86)***	−7.90 (− 9.88; − 5.93)***	5.58 (4.15; 7.02)***
	Resilience training	− 15.14 (− 17.70; − 12.59)***	−8.67 (− 10.61; − 6.72)***	6.48 (4.60; 8.36)***

*P value< 0.05; **P value< 0.01; ***P value< 0.001

In all cases, the second time is minus the previous time

*CI* Confidence Interval

LSD post hoc test was performed

According to the between-groups comparison results, concerning WFC and its domains, there was no statistically significant difference in the mean pretest scores among the three groups. The mean scores for the post-test of WFC and its domains (excluding inadequate facilities and support) demonstrated a statistically significant difference among the three groups. The mean scores for the follow-up of WFC and its work role interference with personal and family life domain showed a statistically significant difference among the three groups (Table 5). Two-by-two comparisons showed that the mean scores for the post-test of WFC and its domains (excluding inadequate facilities and support) were similar in the two intervention groups and lower than the control group. The mean scores for the follow-up of WFC and work role interference with personal and family life domain were similar in the two intervention groups and lower than the control group. In other domains, the mean score of the follow-up among the three groups was not statistically significant (Table 7).

**Table 7:** Mean difference and confidence interval for two-by-two comparison of work-family conflict and its domains between three groups of time management training, resilience training and control

Variable	Group	Time	
		Post-test	Follow-up
		Mean difference (95% CI)	Mean difference (95% CI)
Work role interference with personal and family life	Time management with Resilience	2.60 (−4.84; 10.04)^NS^	0.82 (−7.00; 8.64)^NS^
	Time management with Control	−14.92 (− 21.79; −8.06)***	−10.91 (− 18.12; − 3.70)**
	Resilience with Control	−17.53 (− 24.45; − 10.60)***	− 11.73 (− 19.01; − 4.45)**
Family dissatisfaction	Time management with Resilience	−2.048 (− 9.85; 5.75) ^NS^	The comparison of three groups was not statistically significant
	Time management with Control	−9.909 (− 17.10; − 2.72)**	
	Resilience with Control	− 7.861 (− 15.12; − 5.61)**	
Family role interference with work role	Time management with Resilience	− 0.96 (− 12.69; 10.76) ^NS^	The comparison of three groups was not statistically significant
	Time management with Control	−12.45 (− 20.26;- 8.64)**	
	Resilience with Control	−11.49 (− 20.40; − 7.58)**	
Total work-family conflict	Time management with Resilience	0.46 (− 4.13; 5.04) ^NS^	−0.44 (− 5.08; 4.21) ^NS^
	Time management with Control	− 8.56 (− 12.79; − 4.33)***	− 5.45 (− 9.74; − 1.17)*
	Resilience with Control	− 9.02 (− 13.28; 4.75)***	−5.02 (− 9.34; − 1.85)*

*P value< 0.05; **P value< 0.01; ***P value< 0.001

In all cases, the first group is minus the second group

CI=Confidence Interval; NS=Non-significant

LSD post hoc test was performed

## Discussion

The objective of this study was to examine the impact of time management and resilience training on WFC among Iranian female nurses. The results revealed that the mean posttest and follow-up score of WFC was similar in both intervention groups and lower than the control group. However, the average WFC score increased from posttest to follow-up in all the three groups. In conclusion, the results indicate a nearly similar effect of the two interventions on reducing WFC. Two interventions had no impact on the inadequate facilities and support domain. As a result, all three research hypotheses are almost supported.

The present study’s findings regarding the impact of time management on WFC is consistent with the study of Rasooli et al. (2009) and Khosravan et al. (2018) on Iranian nurses as well as the Ebrahimi Aval et al. (2022) on hospital emergency technicians in Iran [18, 19, 39].

Noroozi et al. (2022) found that multimedia training reduced WFC among Iranian women employed in healthcare services [40]. They also reported that the multimedia training increased the support by the spouse and officials in workplaces. These findings are contrary to the present study where the two interventions did not affect the areas of inadequate facilities and support. This discrepancy could be attributed to the type of training programs, as the present study focused on individual-based interventions, while Noroozi et al. included training for spouses and officials.

About impact of resilience intervention on WFC, consist with present study, Ahmadi et al. (2019) demonstrated that resilience improves the quality of work life among Iranian nurses. Quality of work life involves problem-solving skills, indicating that resilience training could help individuals better address personal problems [29].

Since both mindfulness and resilience interventions reduce stress, it is reasonable to compare their outcomes. In study by Kiburz et al. (2017), the mindfulness intervention decreased work-to-family interference, but did not reduce family-to-work conflict among university and community college employee. In present study resilience intervention reduce both sides of the WFC. The researchers cited the floor effect as the reason for the intervention’s lack of impact on family-work interference in the mentioned study. The difference in the measures used or the smaller sample size in Kiburz’s study may be another reason for the difference in results between the two studies.

Chen et al. (2021) reported a negative statistical relationship between WFC and resilience among Chinese nurses, which is in alignment with the present study [41]. Studies emphasizing psychological capital, particularly resilience, have found a negative statistical relationship with various types of WFC, consistent with the current findings [22, 42].

Regarding the third hypothesis of the study, which explores the similarity of the impact of two interventions on reducing WFC, the findings demonstrate a similar impact of both interventions.

No study was found that directly compared the impact of both interventions on reducing WFC. In a qualitative study of university teachers in Australia and Germany, the researchers found that effective time management and resilience play critical roles in managing work-family challenges [43]. A study conducted in Sri Lanka revealed that resilience is positively correlated with planning and leadership skills among nurses [44]. These results indirectly support the findings of the present study.

According the Greenhouse and Beutel’s theory, pressure and time emerge as crucial factors influencing WFC [13]. Alternatively, drawing from the Conservation of Resources Theory (Hobfoll, 1989), [14] Resilience and time management, perceived as a learnable personal resource, functions as a coping mechanism to alleviate stress, reinforcing the capacity to navigate the complexities of work-family dynamics.

Effective time management allows individuals, particularly women in the context of this study, to establish clear boundaries between work and family. By managing time efficiently and prioritizing tasks, women can alleviate the burden of workload overflow, contributing to increased satisfaction in familial roles such as wife or mother and consequently decreasing family dissatisfaction. Moreover, effective time management enables working women to allocate time for self-care, leading to a reduction in personal complications arising from the challenges of juggling dual roles.

In the context of resilience training, the acquisition of traits such as hope, faith, purposefulness, altruism, empathy, self-control, optimism, emotional intelligence, flexibility, and adaptability comprises the components of resilience training [29], enable healthcare professionals to navigate tensions and conflicts more adeptly, fostering emotional resilience in the face of work-family challenges.

The control group’s average scores for work role interference with personal and family life and family dissatisfaction domains were lower in the post-test and follow-up than in the pre-test, with a slight increase in the follow-up. This could be attributed to a pre-test effect, where awareness of WFC areas prompted control group participants to take initial actions [45].

The average score for WFC during the follow-up was higher than the post-test, indicating a reduction in the intervention’s effectiveness. This differs from Ebrahimi Aval’s study where the follow-up score was lower than the post-test [39]. The discrepancy may be due to the intervention duration and data collection timing differences. The present study spanned more than 2 months, whereas Ebrahimi Aval’s intervention lasted 1 day, with data collected immediately and 1 month later. A systematic review in the Cochrane Database suggested that the effect of resilience intervention was significant in the follow-up of less than 3 months, but not significant in the long-term follow-up, meaning that the effect of resilience interventions diminishes over time [46], hence emphasizing the need for repeated interventions to maintain effectiveness.

In conclusion, the study suggests a comparable effect of time management and resilience training on reducing WFC among female nurses. However, due to the small sample size, especially in the intervention groups, the confidence intervals for mean differences were relatively wide, indicating the need for larger studies to validate the results. Replicating interventions and testing various approaches can further refine our understanding of effective strategies to reduce WFC.

### Application of results and recommendations for future studies

Based on the study results, planning time management and resilience training for nurses is a significant step in reducing WFC in this group. Health systems and organizations interested in addressing WFC among nurses can benefit from designing regular training courses on time management and resilience. Hospitals can include these courses in their continuous training programs, inviting experts and conducting regular intervals of training. The reduction of WFC contributes to improved fulfillment of family roles and job duties, ultimately leading to decreased stress and increased life and job satisfaction for female nurses. However, it is crucial to note that educational interventions focusing solely on the individual may not be sufficient to optimally reduce all aspects of WFC. Additional organizational and family measures and interventions are deemed essential. Training spouses, officials, and colleagues can broaden the scope of support and facilities, enhancing the overall impact of interventions. Conducting further interventions and comparing their efficacy can help determine the most effective measures in reducing WFC among nurses.

### Limitations of the study

Despite the positive results, there are several limitations to consider. The relatively small sample size and dropouts in both intervention groups may impact the study’s findings. This can be addressed by examining larger study sample sizes for increased statistical power. The lack of blinding due to the study design introduces potential bias. Additionally, the study’s focus on nurses in public hospitals may limit the generalizability of results to nurses in private units. The exclusive use of quantitative data collection and analysis methods is another limitation, suggesting that future studies may benefit from a mixed-methods approach for a more comprehensive understanding and mitigating potential methodological biases.

## Conclusion

The study findings indicate that both time management and resilience training similarly reduce the mean score of WFC in the post-test and follow-up compared to the pre-test. However, the average score during follow-up was higher than in the post-test, and the domain of inadequate facilities and support was not significantly affected. In summary, time management and resilience training can be considered effective interventions in continuous training programs for nurses. The diminishing effect over time of these training sessions suggests the importance of ongoing training. Further research and exploration of alternative interventions are necessary to identify the best measures for optimal WFC management among nurses.

## Data Availability

The datasets used during the current study are available from the corresponding author on reasonable request.

## Abbreviations

ANOVA: Analysis of variance
GLM: General linear model
LSD: Least Significant Difference
MS: Master
SPSS: Statistical Package for the Social Sciences
WFC: Work-family conflict
